# Experimental Study of a Broadband Vibration Energy Harvester Based on Orthogonal Magnetically Coupled Double Cantilever Beam

**DOI:** 10.3390/mi16060722

**Published:** 2025-06-19

**Authors:** Yanhao Feng, Jianhua Wang, Xiangye Chen, Peng Liu

**Affiliations:** College of Aeronautics and Astronautics, Taiyuan University of Technology, Taiyuan 030024, China

**Keywords:** magnetic coupling, orthogonal cantilever beam, vibration energy harvesting, broadband, piezoelectric energy capture, nonlinear resonance control

## Abstract

**Purpose**: The aim of this study is to achieve automated energy capture and charging for the ADXL355 accelerometer, enhance the vibration energy collection efficiency, and widen the energy trapping frequency band of a system in a working environment for bridge health state detection. **Methods**: A vibration energy harvester based on a magnetic coupling cantilever beam in an orthogonal direction was proposed. The harvester works by adjusting the angle and magnetic spacing between the two cantilever-beam piezoelectric oscillators, enabling the oscillators to produce large-scale and stable vibrations when excited by an external broadband vibration source. **Results**: Sinusoidal frequency sweep experiments showed that, under an excitation amplitude of 0.2 g, the proposed broadband vibration energy harvester based on orthogonal magnetic coupling double cantilever beams achieved the best energy harvesting performance when the magnetic angle of the double cantilever beam system was 130°, and the radius was 16 mm. In the frequency range of 5–20 Hz, the system can effectively capture higher effective voltages across all frequency bands, with a total captured voltage value of approximately 15.3 V. Compared with the control group, the system’s energy harvesting capacity under this working condition increases by 770%. Additionally, the effective frequency band of the system was broadened by 3.7 Hz. **Conclusions**: Unlike previous studies, which often limited the angles of the magnetic fields generated by the magnets at the ends of piezoelectric beams to specific values, this study explores the influence of rotating these magnetic fields to general angles on the working frequency band of the structure. The findings provide a new perspective and theoretical basis for the optimal design of broadband vibration energy harvesters.

## 1. Introduction

With the continuous maturation of microelectromechanical systems (MEMS) and electronic technologies, there are four main trends in electronic products: miniaturization, multifunctionality, portability, and low power consumption [1]. Various low-power devices have rapidly permeated people’s daily lives, and most of these devices rely on chemical batteries for power supplies. However, chemical batteries have a short lifespan, high maintenance costs, relatively large sizes, and are likely to cause environmental pollution [2]. With the advancements in energy conversion technologies for diverse energy sources, including solar energy, wind energy, biomass energy, and vibration energy harvesting, research on powering small-scale electronic devices has gained significant traction. For instance, studies have explored photovoltaic film [3], thermoelectric film [4], windsolar hybrid systems [5], and biofuel cells [6]. Among these, the design of vibration-based energy harvesters (VBEHs) has emerged as a prominent focus. Because environmental vibration sources are ubiquitous in many daily applications, scholars have focused on how to harvest energy from them to achieve a self-powered supply for electronic devices [7]. The mechanical energy of the road surface generated by repeated traffic loads and vibrations is one of the most common forms of energy and has a higher energy conversion rate than other environmental energy sources [8].

According to the differences in the energy conversion mechanisms, the devices used for realizing vibration energy harvesting fall into three main categories: electromagnetic [9], electrostatic [10], and piezoelectric [11]. Compared to other energy harvesters, piezoelectric vibration energy harvesters generate electrical output under mechanical stress, and this characteristic has been used for energy harvesting in many applications [12]. As an unconventional, feasible, and clean solution, they meet global energy demands and play an important role in powering a variety of portable electronic devices, wireless sensor nodes, and medical implants [2]. Research has shown that when the external excitation frequency is the same as the natural frequency of the piezoelectric energy harvester, the energy conversion efficiency of the system is highest. In practical applications, owing to the characteristics of a wide frequency band and the randomness of the vibration frequencies in the external environment, when the natural frequency of the piezoelectric energy harvester cannot always match the external excitation frequency, its power generation performance is greatly limited [13]. Therefore, a piezoelectric energy harvester must have high energy conversion efficiency within a large frequency band range. Currently, methods for realizing wideband vibration energy harvesting include [14], tuning techniques [15,16], techniques exploiting nonlinear oscillations [17], multimodal energy harvesting [18,19], and frequency up-conversion [20,21]. Although these methods exhibit distinct advantages and drawbacks, the magnetic nonlinear approach stands out for its relatively abundant research corpus.

Systems that harvest energy using the magnetic nonlinear method are divided into vibration energy harvesters with nonlinear monostable and bistable characteristics. For vibration energy harvesters with nonlinear monostability, Stanton et al. [22] modeled and experimentally validated a nonlinear energy harvester capable of bidirectional hysteresis. Both hardening and softening responses within the quadratic potential field of a power-generating piezoelectric beam (with a permanent-magnet end mass) were invoked by tuning the nonlinear magnetic interactions. Not only is this technique shown to increase the bandwidth of the device, but the experimental results also verify its capability to outperform linear resonance. Engaging in this nonlinear phenomenon is ideally suited to efficiently harvesting energy from ambient excitations with slowly varying frequencies. Xing et al. [23] proposed a vibration energy harvester based on a cantilever beam with a high magnetic permeability. The coexistence of magnetostatic and elastic potential energies results in nonlinear oscillations with a wide bandwidth. For vibration energy harvesters with nonlinear bistability, Cottone et al. [24] demonstrated that a bistable oscillator under proper operating conditions can provide better performance than a linear oscillator in terms of the energy extracted from a generic wide-spectrum vibration. Erturk et al. [25] developed a non-resonant piezomagnetoelastic energy harvester. Following theoretical simulations, it was experimentally verified that the non-resonant piezomagnetoelastic configuration could generate an order of magnitude larger power than the commonly employed resonant piezoelastic configuration at several frequencies. Lan et al. [26] developed an improved bistable PEH mechanism by adding a small magnet between the two fixed magnets. It has been proven that the attractive force from the additional magnet can effectively minimize the potential and enable the device to easily achieve a snap-through jump, even under weak excitation. Su et al. [27] proposed a broadband magnet-induced dual-cantilever piezoelectric energy harvester. The dual-cantilever structure consists of outer and inner beams with magnets attached to the tips. The magnets generated a nonlinear repulsive force between the two beams and rendered the structure bistable. The simulation and experimental results showed good agreement with respect to the power bandwidth and amplitude.

The above research results show that compared with other structures that concentrate on broadening the operating frequency band of the system, the bistable system has a more remarkable effect, but it also has certain defects. First, when the externally applied excitation frequency deviates from the operating frequency band of the system, the output voltage of the system is extremely small and even smaller than the output amplitude of the linear system. The bistable system exhibits high energy-harvesting efficiency only when the end mass of the cantilever beam makes a stable large-amplitude transition movement across the two stable states. In other words, only when the movement of the end mass of the bistable system breaks through a certain threshold can the system perform stable bistable movements [28]. To broaden the operating frequency band of a bistable system and improve its adaptability to the excitation of the vibrational environment, Zhou et al. [29] investigated a magnetically coupled nonlinear piezoelectric energy harvester by altering the angular orientation of its external magnets to enhance the broadband frequency response. The nonlinear energy harvester proposed in this study can cover a broad low-frequency range (4–22 Hz) by changing the magnet orientation. Zou et al. [30] designed a magnetically coupled vibration-energy harvester with two degrees of freedom in rotational motion. This device was designed for low-speed rotation and can harvest vibrational energy in various frequency bands. Guan et al. [31] proposed a piezoelectric energy harvester for rotational motion applications. The piezoelectric energy harvester was installed on a rotational system in which the rotational axis was approximately parallel to the Earth’s surface. When the harvester rotates, its piezoelectric elements are repeatedly deformed to generate electrical energy. Yin et al. [32] proposed a rolling-swing electromagnetic energy harvester (RS-EMEH) that creates the counter-rotations between magnets and coils, thereby amplifying the magnetic field variation within a single cycle of ultra-low-frequency excitation.

While using the magnetic nonlinear method to broaden the operating frequency band, researchers have aimed to improve the system’s ability to collect multi-directional vibrations by changing the number and direction of the beams. Andò et al. [33] proposed a suitable topology of the double bistable oscillator to obtain a bi-axial vibrations energy harvester. The system proposed here is composed of two magnetically coupled bi-stable beams, each with orthogonal directions of deflection and piezoelectric output. In the presence of mechanical vibrations, the beams will respond to both the inertial excitation and the magnetic coupling. Su et al. [34] proposed a three-directional broadband vibration energy harvester. This harvester is composed of a main beam, auxiliary beam, and spring-mass system, which are used to introduce nonlinear forces and couple the three subsystems to realize the collection of multi-directional vibrations. Jackson [35] investigated three different magnetic configurations and their effects on bandwidth and power generation by using attractive and repulsive magnetic forces. The study also involved the development of a piezoMEMS device for harvesting vibration energy, which monolithically integrates a thick embedded permanent magnet (NdFeB) film. Chen et al. [36] developed a sequential rotary-driven piezoelectric wave energy harvester. A one-way bearing was used to convert the repeated rotational motion of the input shaft into one-way rotational motion without reverse rotation to drive the rotational piezoelectric harvesting component and effectively reduce the damage risk of the piezoelectric composite cantilever beam structure.

In addition, researchers have also considered replacing magnets with ferromagnetic fluids, thereby transmitting energy through the flow and deformation of the ferromagnetic fluids. Maskar et al. [37] designed a suspension system that combines an MR fluid damper and an energy-harvesting rack and pinion system. Soheil et al. [38] investigated the energy harvesting from the vibrations of an axially moving circular cylindrical composite nanoshell conveying an internal nanofluid flow for the first time, considering the system under induced vibration.

However, in previous related studies, the included angles of the magnetic fields generated by the magnets at the ends of the piezoelectric beams were all limited to specific angles, such as 180°. To date, there has been no in-depth exploration of the influence of the magnetic fields of the magnets at the ends of piezoelectric beams rotating to a general angle on the operating frequency band of the structure. This paper proposes a biaxial piezoelectric beam bistable vibration energy harvester based on magnetic coupling. By systematically changing the included angle between the piezoelectric oscillators of the cantilever beams orthogonal in two directions and the magnetic spacing, an in-depth study was conducted on the influence of the operating frequency band of the bistable structure within a specific magnetic spacing range and when the piezoelectric oscillators are at a general angle. This study aims to design a vibration energy harvester capable of stably powering the ADXL355 acceleration sensor (operating voltage range: 2.25–3.6 V), enabling accurate detection of the bridge vibration response to external forces such as vehicle passage, wind loads, or earthquakes.

## 2. Working Principle

**Figure 1:**
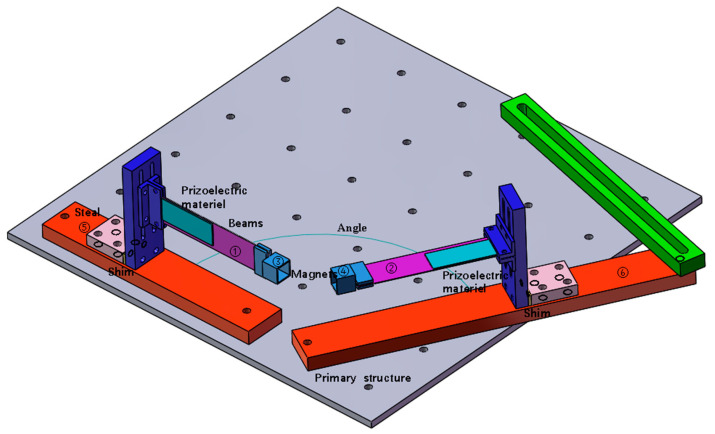
Structure of the vibrational energy harvester.

**Figure 2:**
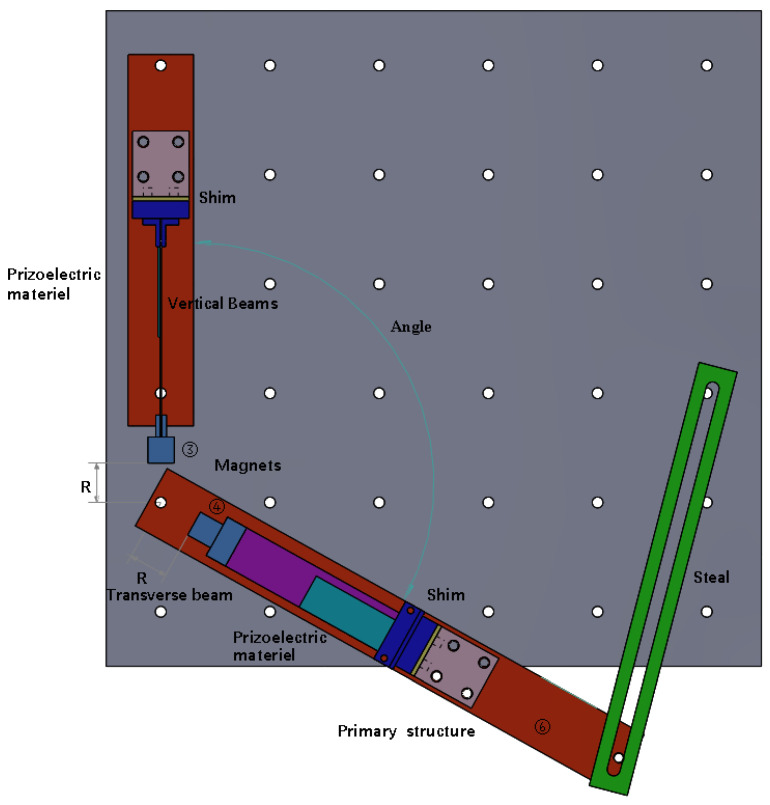
Structure of the vibrational energy harvester (top view).

The structure of a vibration energy harvester is shown in Figure 1 and Figure 2. The working system consisted of two cantilever beams, two magnets, two fixtures for fixing the cantilever beams, two iron plates fixed on the shaking table, and several gaskets for adjusting the magnetic spacing. Number of beams ①–②, magnets ③–④, and iron plates ⑤–⑥. The magnets were fixed at the ends of the two cantilever beams, and the repulsive poles were placed opposite to each other. The repulsive force generated by the two magnets at an appropriate distance caused the entire beam system to vibrate nonlinearly. The system exhibited bistable or monostable behavior in different frequency ranges. This phenomenon is due to the difference between the magnetic angle and the magnetic radius, resulting in different magnetic coupling effects. The working principle is that when the structure is installed on the shaking table, as the shaking table produces transverse excitation, the cantilever beam produces a response perpendicular to the respective beam surface, and the piezoelectric sheet that drives the adhesion to the beam produces deformation. According to the positive piezoelectric effect, the upper and lower ends of the piezoelectric sheet will gather positive and negative opposite charges and then connect with the external circuit to generate the output current. Under magnetic coupling, the cantilever beam produces vibration caused by the excitation of a single beam and the nonlinear vibration of the double beam owing to the coupling effect of the double beam. In this case, even if the excitation frequency can only cause one of the beams to vibrate, the other beam will vibrate owing to magnetic coupling. Beam ① captures the transverse vibration energy in the same direction as the excitation direction of the shaking table, and beam ② captures the longitudinal vibration energy perpendicular to the excitation direction. By adjusting the angle and magnetic spacing between the two magnets, the energy-trapping characteristics under the double-girder structure are displayed to increase the energy-trapping effect at a specific frequency and widen the frequency band.

## 3. Experimental Setup and Methodology

### 3.1. Experimental Setup

**Figure 3:**
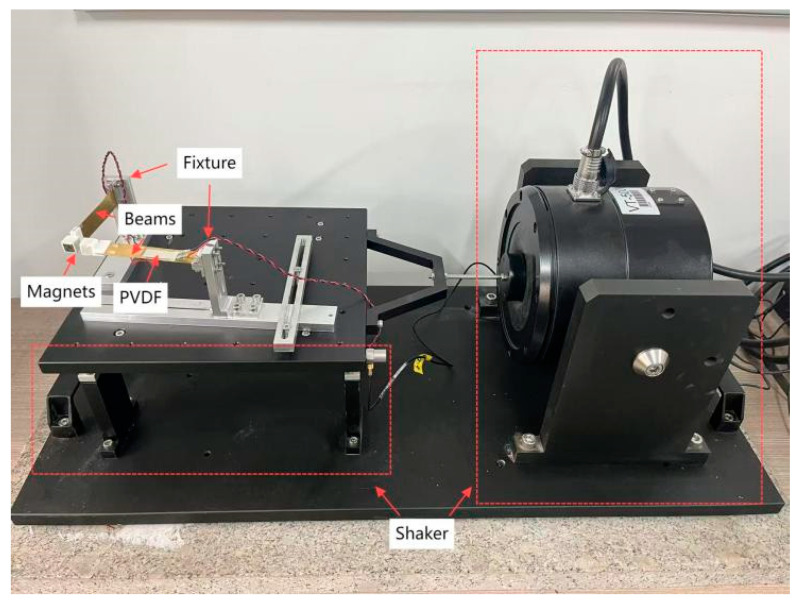
Prototype of the PEH proposed in this study.

**Figure 4:**
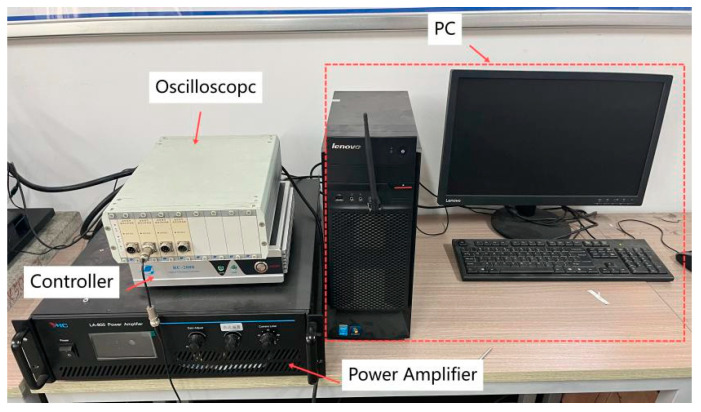
Experimental setup.

The physical model and experimental setup of the double girder are illustrated in Figure 3 and Figure 4. The cantilever beam was made of brass material, and the cantilever beam was firmly fixed in the clamp structure by drilling holes in its corresponding position with the fixture structure, penetrating the bolts that fit the specifications, using the tool to tighten the nut, and relying on the extrusion force generated by bolt tightening. The magnet was placed in a 3D-printed magnet seat at the opposite polar position and fixed to the end of the cantilever beam. The internal parts of the fixture structure were matched using a threaded connection, with the matching threads processed on the corresponding parts and then tightly screwed together. The fixture and shaker table were firmly fixed on the shaker table by drilling holes in the corresponding positions using adaptation bolts to penetrate and tighten the nuts. Beam ① was placed perpendicular to the excitation direction, and beam ② was placed parallel to the excitation direction. With the excitation of the shaking table, the piezoelectric sheet captured the energy generated by the respective beam. The piezoelectric sheet is a thin-film piezoelectric sheet of the LDT1-028K model. The magnet is of the type Nd35.

To verify the feasibility of reversing the excitation piezoelectric vibration trap and the relationship between its output performance and related parameters, the test prototype and test system shown in Figure 3 were designed and fabricated. The main instruments were a YMC LA-800 power amplifier (Yangzhou Yingmaike Measurement & Control Technology Co., Ltd., Yangzhou, China), VT500 shaker (Yangzhou Yingmaike Measurement & Control Technology Co., Ltd., Yangzhou, China, working frequency of 5–20 Hz, maximum acceleration of 0.2 g), shaker controller (RC2000, Yangzhou Yingmaike Measurement & Control Technology Co., Ltd., Yangzhou, China), and DHDAS digital acquisition instrument (Jiangsu Donghua Testing Technology Co., Ltd., Jingjiang, China).The excitation signal is sinusoidal Table 1. Parameters of manufacturing physical models list the parameters of the vibration pickup, transducer, piezoelectric oscillator, and magnet. The output voltages mentioned in the experiments were open-circuit voltages measured directly after the piezoelectric oscillators were electrically parallel.

### 3.2. Experimental Methods

The experimental conditions were as follows: the shaking table generated a horizontal excitation, and the double girder was placed in an orthogonal form. Because we wanted to explore the influence of magnetic coupling on the energy-trapping effect of the structure, we could only consider the experimental results of the shaking table vibrating in one direction. Two sets of variables were set up in the experiment. One group was designed with the intersection point of the two beam axes as the center of the circle, the iron plate was fixed, the angle between the iron plates in the structure was changed by rotating the iron plate to change the magnetic angle, and the angle was regarded as the angle variable of the experiment. In view of the fact that the magnetic spacing between the two magnets will be significantly shortened when the magnetic angle between the two magnets is at an acute angle, this phenomenon may lead to the occurrence of physical collision due to the model design on the one hand, and on the other hand, the magnetic effect of the edge of the magnet will be significantly enhanced, resulting in the adsorption effect between the side of the magnet ③ and the magnet ④, which will affect the experimental results. Therefore, the angle setting of this experimental setup began at a right angle of 90°. There are ten sets of angles in this variable group, which are 90°, 100°, 110°, 120°, 130°, 140°, 150°, 160°, 170°, and 180°. In the other set of designs, the distance from the end of the beam to the center of the circle was changed by changing the number of spacers between the clamp connected at the end of the cantilever beam and the fixed block on the foundation device. The magnetic spacing was changed, and the distance was regarded as the radius variable of the experiment. The variable group was set to four radii: 14 mm, 16 mm, 18 mm, and 20 mm. A control group was set up, and the same experimental process was performed by replacing the magnetic block with a mass of the same mass. The results were compared with those of the variable group to determine the effect of magnetic coupling.

In the entire experiment, transverse excitation was applied in the form of a sine sweep; the starting frequency was 5 Hz, the end frequency was 20 Hz, the acceleration peak was 0.2 g, and the swept frequency was 2 Hz. The effect of the magnetic force on the beam under different variables in the experiment is reflected in the amplitude of the vibration of the beam, and its response interval to the external frequency resonance; the effect of magnetic coupling is presented as a voltage-time image. Considering that the bridge generates low-frequency vibrations under traffic loads or natural wind vibrations, we designed the energy-trapping structure to supply power to the ADXL355 accelerometer (Analog Devices, Wilmington, MA, USA) with a working voltage in the range of 2.25 to 3.6 V so as to achieve the automation of autonomous energy capture and charging of the structural system. Therefore, we defined the effective bandwidth of the continuous segment with a voltage value of more than 3 V and defined the consolidation of the segment with an adjacent peak spacing of <10% of the fundamental frequency as the unified bandwidth. The energy-trapping efficiency of the structure is expressed by calculating and comparing the RMS value under the bandwidth, where the RMS value is calculated using the following formula:

(1) $$V_{\mathrm{rms}}=\sqrt{\frac{1}{f_{2}-f_{1}}\int_{f_{1}}^{f_{2}}v^{2}(f)df}$$

## 4. Experimental Results

### 4.1. Raw Data Display

**Figure 5:**
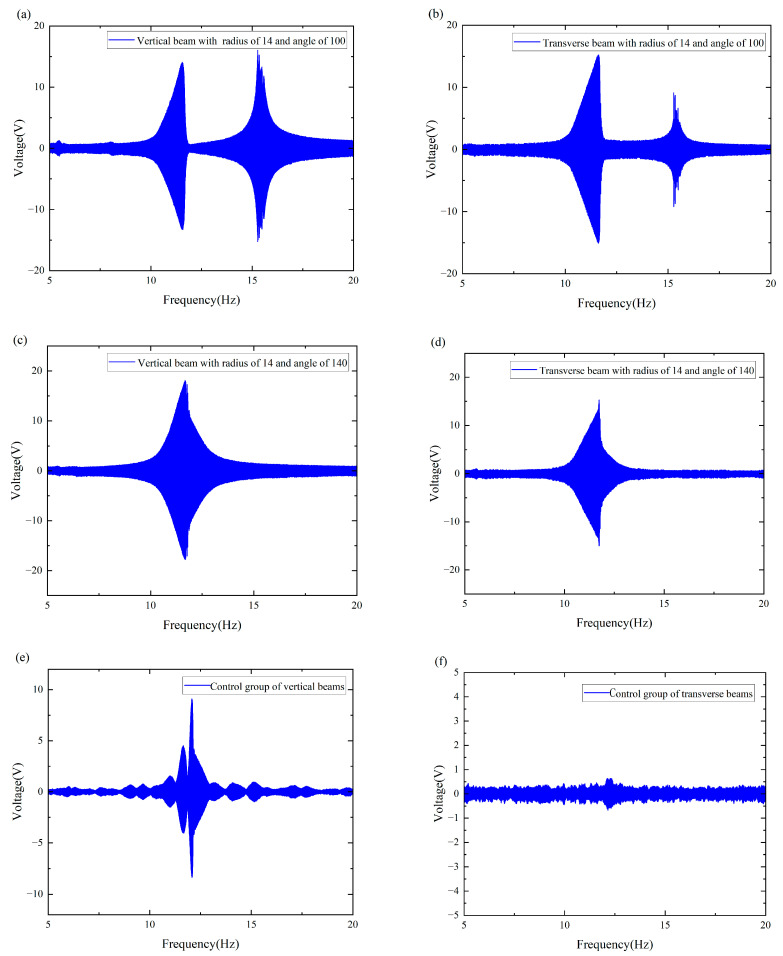
The raw data images: (**a**) The vertical beam with a radius of 14 and an angle of 100. (**b**) The transverse beam with a radius of 14 and an angle of 100. (**c**) The vertical beam with a radius of 14 and an angle of 140. (**d**) The transverse beam with a radius of 14 and an angle of 140. (**e**) The vertical beam of the control group. (**f**) The transverse beam of the control group.

The raw data images are shown in Figure 5a–f, which represent the different trapping effects owing to changes in external environmental frequencies during our experiments. As can be seen from Figure 5a,b, the double girder exhibits a large abrupt change around 10–12 Hz and a second peak at 15–17 Hz. As can be seen from Figure 5c,d, the main resonance peak of the system shifts and is concentrated between 11 and 13 Hz under the change in angle. Moreover, the two peaks merge at 12 Hz. As shown in Figure 5e,f, the natural frequency of the beam was approximately 12 Hz. Since the control group did not exhibit a magnetic coupling effect, changing the variable had no effect on its response. These phenomena show that both the angle and distance affect the energy trapping effect of the system; therefore, it is very meaningful to explore the optimal angle and distance of the double girder in this interval to improve the frequency trapping efficiency of the entire system and achieve a widening of the frequency band.

### 4.2. Data Processing

**Figure 6:**
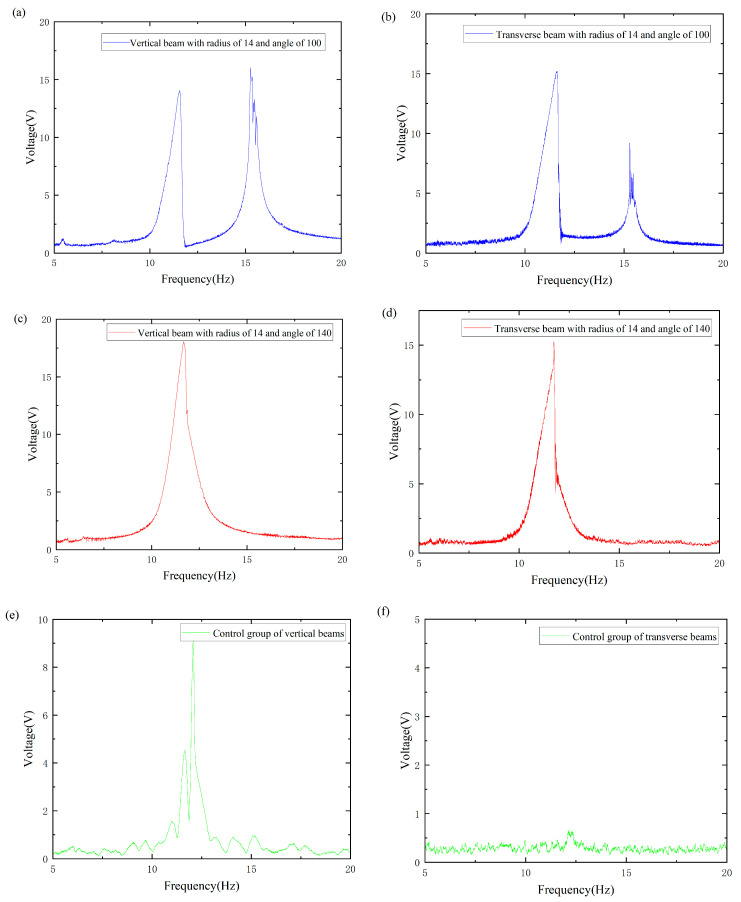
The processed image: (**a**) Image of a vertical beam with radius of 14 and an angle of 100. (**b**) Image of a transverse beam with radius of 14 and angle of 100. (**c**) Image of a vertical beam with radius of 14 and angle of 140. (**d**) Image of a transverse beam with radius of 14 and angle of 140. (**e**) Images of the control group of vertical beams. (**f**) Images of the control group of transverse beams.

To enhance the contrast of the overall image, the original harmonic image was processed into a smooth envelope using MATLAB2021. The processed images are shown in Figure 6a–f. Subsequently, the length of the defined bandwidth and the effective value of the envelope within each effective bandwidth were calculated. A nonlinear surface-fitting method was used to predict the optimal radius and angle by comparing the effective values obtained by MATLAB processing.

### 4.3. Effect of Magnetic Angle on the Performance of the Collector System

**Figure 7:**
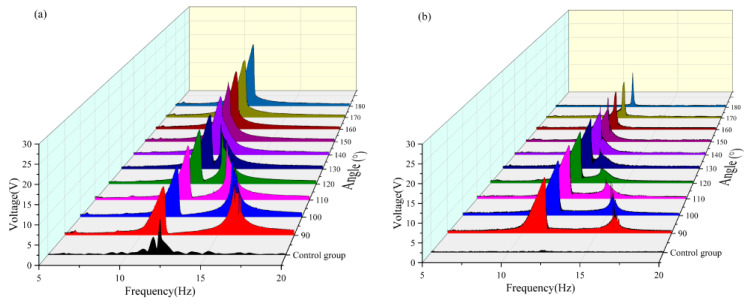
(**a**) Comparison images of vertical beams with a radius of 14 mm and variable angles. (**b**) Comparison images of transverse beams with a radius of 14 mm and variable angles.

The angle between the magnets was changed to analyze the output voltage and bandwidth captured by the system, as shown in Figure 7. The results indicate that, as the angle gradually increases from 90° to 180°, the frequency at which the second resonance peak of the system appears shifts progressively forward and merges with the first resonance peak near 12.5 Hz. When the magnetic angle reached 180°, the second resonance peak was fully integrated with the first resonance peak, resulting in a single resonance characteristic. This is due to the fact that when the angle between the magnets increases from 90° to 180°, the enhancement of the axial repulsion component of the magnetic field reduces the equivalent stiffness of the system and promotes the bistable transition and modal coupling, which ultimately leads to the shift and widening of the resonance range to the low-frequency band. This phenomenon provides a key design basis for optimizing the two-girder energy-trapping system. By adjusting the magnet angle, the energy-harvesting efficiency can be maximized in a specific frequency band.

As shown in Figure 7. With the variation in the angle between the magnets, the resonance frequencies of the first main peaks of the two beams were all within the range of 10–12.5 Hz. The second main vibration peak approached the first main peak continuously as the angle increased. When the magnetic angle reached 140°, the double peaks merged, and the response interval of the main vibration peak continued to decrease. When the magnetic angle is 180°, the response interval of the main vibration peak of the transverse beam has been compressed to approximately 0.5 Hz, and the peak intensity is significantly reduced. In contrast, the amplitude of the main vibration peak of the vertical beam shows an increasing trend, which is attributed to the magnetic field symmetry reconstruction at a 180° magnetic angle that alters the dynamic behavior of the system. For the transverse beam, the enhanced orthogonal coupling damping reduced nonlinearity, and shallower potential wells compress the response bandwidth and diminish the peak amplitudes. Conversely, the vertical beam benefits from optimized energy transfer pathways, augmented nonlinear stiffness, and mitigated modal competition, leading to a notable increase in the main resonance amplitude. These findings underscore the pivotal role of geometric parameters in governing the energy distribution within multistable systems, offering critical insights for the targeted control of vibration energy harvesting.

The effective voltage values captured at each angle with a radius of 14 mm are compared in Table 2 and Figure 7. It can be seen that when the radius is 14 mm, the effective voltage is not captured by the transverse and vertical beams in the frequency band of 5–10 Hz. Within the frequency band of 5–10 Hz, a tiny voltage output can be observed only when the magnetic angle of the vertical beam is in the range of 170–180° can a tiny voltage output be observed. In the frequency band of 10–15 Hz, the vertical beam reached its maximum voltage output (approximately 7.466 V) when the magnetic angle was 180°, whereas the output of the crossbeam was only approximately 1.778 V at this moment. Conversely, the crossbeam reached its maximum voltage (approximately 5.638 V) when the magnetic angle was 110°, and the corresponding voltage output of the vertical beam was approximately 6.639 V. In the frequency band of 15–20 Hz, both the vertical and cross beams achieve effective energy harvesting within the magnetic angle range of 90–110°, reaching the voltage peak when the magnetic angle is 100°. A comprehensive analysis reveals that the system exhibits optimal energy-harvesting efficiency when the magnetic angle is 100°. Under this condition, the effective frequency band of the vertical beam was broadened by approximately 1 Hz, and that of the crossbeam was broadened by approximately 2.5 Hz.

**Figure 8:**
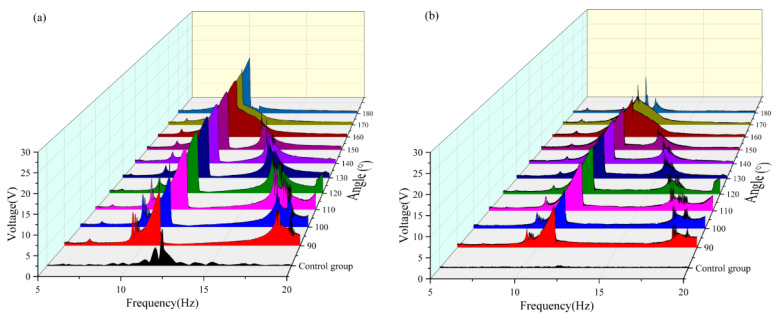
(**a**) Comparison images of vertical beams with a radius of 16 mm and variable angles. (**b**) Comparison images of transverse beams with a radius of 16 mm and variable angles.

The effective voltage values obtained at each angle are compared in Table 3 and Figure 8. It can be observed that when the radius is 16 mm in the frequency band of 5–10 Hz, the vertical beam reaches the maximum effective voltage output (approximately 3.529 V) when the magnetic angle is 180°, during which the crossbeam fails to capture any effective voltage. Conversely, the crossbeam achieved the maximum effective voltage when the magnetic angle was 150°, with a corresponding output of the vertical beam of 3.1 V. In the frequency band of 10–15 Hz, both the vertical and cross beams achieved a peak output of the effective voltage when the magnetic angle was 160°. In the frequency band of 15–20 Hz, both beams generated effective voltage only within the magnetic angle range of 90–150°, and both reached the maximum value when the magnetic angle was 120°. By comprehensively analyzing the energy-harvesting performance in each frequency band, it was found that the system had the optimal energy-harvesting efficiency when the magnetic angle was 130°. Under this condition, the effective frequency bands of the vertical beam and crossbeam were broadened by approximately 1.7 Hz and 2 Hz, respectively.

**Figure 9:**
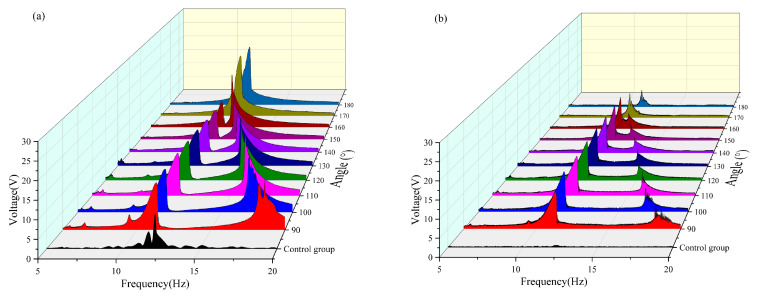
(**a**) Comparison image of a vertical beam with a radius of 18 mm with variable angle. (**b**) Comparison image of a transverse beam with a radius of 18 mm with variable angle.

As shown in Table 4 and Figure 9, when the radius is 18 mm in the 5–10 Hz frequency band, the vertical beam generates a relatively small effective voltage value only when the magnetic angle is 90°; no effective voltage is captured by either beam under other magnetic angle conditions. In the 10–15 Hz frequency band, the vertical beam reached its maximum effective voltage output of approximately 7.268 V at a magnetic angle of 170°, whereas the crossbeam captured a voltage of approximately 1.883 V. Conversely, the transverse beam achieved its maximum effective voltage of approximately 3.928 V at a magnetic angle of 130°, with the corresponding voltage captured by the vertical beam being 3.220 V. In the 15–20 Hz frequency band, both beams capture higher voltage values within the magnetic angle range of 90–120°, and energy harvesting reaches its peak at a magnetic angle of 120°. Based on the performance across all frequency bands, a system with a radius of 18 mm achieved optimal energy harvesting efficiency at a magnetic angle of 90°. Under this condition, the effective frequency band of the vertical beam was broadened by approximately 1.5 Hz, and that of the crossbeam was extended by approximately 2 Hz.

**Figure 10:**
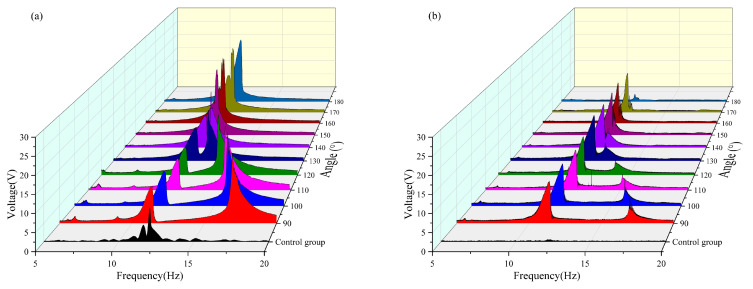
(**a**) Comparison image of a vertical beam with a radius of 20 mm with variable angle. (**b**) Comparison image of a transverse beam with a radius of 20 mm with variable angle.

As shown in Table 5 and Figure 10, when the radius is 20 mm In the 5–10 Hz frequency band, neither the vertical nor the crossbeam achieved effective voltage capture. In the 10–15 Hz frequency band, the vertical beam reached its maximum voltage output of 7.721 V at a magnetic angle of 180° with no voltage response from the crossbeam. Conversely, the transverse beam achieved its peak voltage at a magnetic angle of 130°, with the corresponding output of the vertical beam measured at 6.043 V. Within the 15–20 Hz frequency band, the maximum integrated energy capture was observed for both beams at a magnetic angle of 100°. Through a comprehensive assessment of multiband performance, it was determined that a magnetic angle of 100° represents the optimal configuration for energy harvesting in a system with a radius of 20 mm. Under this condition, the effective bandwidths of the vertical and cross beams were extended by approximately 1.5 Hz each, significantly enhancing the wideband energy capture capability of the system.

To sum up, the optimal angles for the energy harvesting effect of the device under different radius conditions are 90°, 100°, and 130°, respectively. The main reasons for this result are shown in Table 6.

### 4.4. Effect of Magnetic Spacing on the Performance of the Collector System

According to the analysis results of the influence of the magnetic inclusion angle on the energy trapping of the system, the energy trapping efficiency of the system was the best when the magnetic angle of each radius was 90°, 100°, and 130°. Next, the influence of changing the spacing of the magnets on the system trapping energy under these three angles was analyzed to obtain the most suitable state for the trapping energy of the double-girder structure in the experimental group.

**Figure 11:**
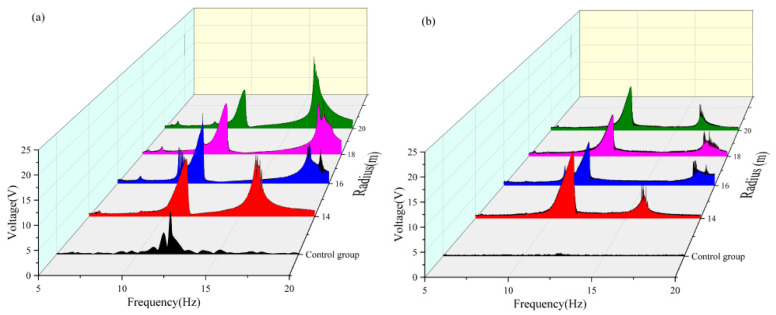
(**a**) Comparative image of a vertical beam with an angle of 90 variable radius. (**b**) Comparative image of a transverse beam with an angle of 90 variable radius.

As shown in Table 7 and Figure 11, when the magnetic angle was 90° in the 5–10 Hz frequency band, both beams achieved the maximum voltage capture value at a radius of 16 mm. In the 10–15 Hz frequency band, the vertical beam attained a maximum voltage capture of approximately 4.414 V at a radius of 16 mm, whereas the transverse beam captured a substantial voltage of approximately 5.21 V at a radius of 14 mm. In the 15–20 Hz frequency band, the vertical beam reached a peak voltage of approximately 6.248 V at a radius of 20 mm. In contrast, the voltage capture values of the transverse beam exhibit negligible variations across different radii, and a comparative analysis of the energy-harvesting performance under varying radii reveals that a radius of 20 mm is more suitable for high-frequency operations, whereas a radius of 16 mm enables both beams to achieve favorable voltage capture across all frequency bands. A comprehensive analysis indicated that when the magnetic angle was 90°, the dual-beam system achieved optimal energy-harvesting efficiency at a radius of 16 mm. Under this condition, the effective frequency band of the vertical beam was broadened by approximately 1.5 Hz, and that of the transverse beam was extended by approximately 1.5 Hz.

**Figure 12:**
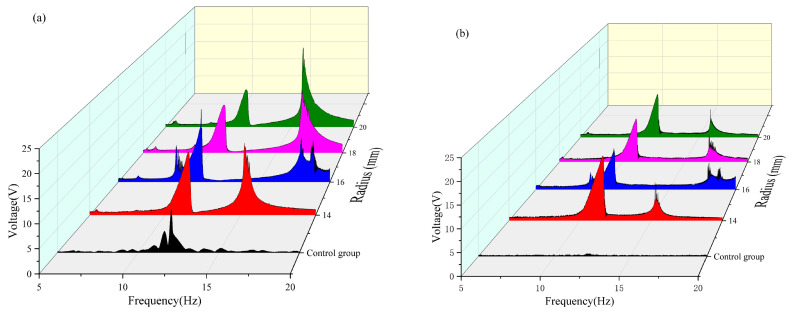
(**a**) Comparative image of a vertical beam with an angle of 100 variable radius. (**b**) Comparative image of a transverse beam with an angle of 100 variable radius.

As shown in Table 8 and Figure 12, when the magnetic angle was set at 100° in the 5–10 Hz frequency band, both the vertical and transverse beams could only capture the effective voltage when the radius was 16 mm. In the 10–15 Hz frequency band, the maximum effective voltage output of both beams was achieved at a radius of 14 mm. In the 15–20 Hz frequency band, the vertical beam reached its peak voltage capture of approximately 6.396 V at a radius of 20 mm, whereas the transverse beam attained its maximum voltage of approximately 1.647 V at a radius of 16 mm. A comparative analysis of the energy-harvesting performance across different radii reveals that a radius of 20 mm is more suitable for high-frequency operations. The dual-beam system with a 16 mm radius demonstrates consistent voltage capture across all frequency bands, whereas the 14 mm radius configuration excels in energy harvesting within the 10–20 Hz band. A comprehensive evaluation of the energy capture performance across all frequency bands indicated that the dual-beam system achieved optimal energy harvesting efficiency at a radius of 14 mm when the magnetic angle was 100°. Under this condition, the effective frequency band of the vertical beam was broadened by approximately 1 Hz, and that of the crossbeam was extended by approximately 2.5 Hz.

**Figure 13:**
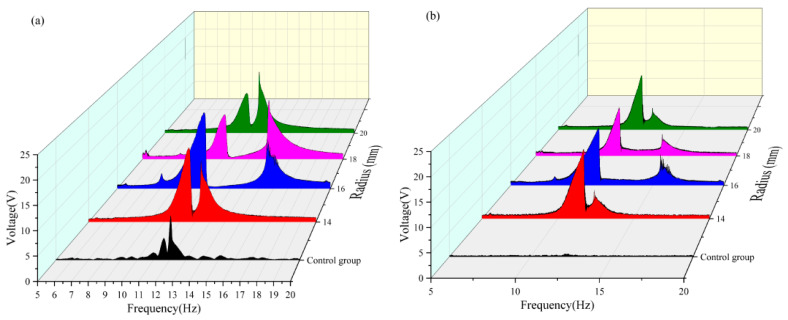
(**a**) Comparative image of the angle 130 variable radius vertical beam. (**b**) Comparative image of the angle 130 variable radius transverse beam.

As shown in Table 9 and Figure 13, when the magnetic angle is 130° in the 5–10 Hz frequency band, both the vertical and transverse beams can only capture the effective voltage values at a radius of 16 mm. In the 10–15 Hz frequency band, the maximum effective voltage output of the dual-beam system was achieved at a radius of 14 mm, whereas in the 15–20 Hz frequency band, the peak effective voltage values were obtained exclusively at a radius of 16 mm. A comparative analysis of the energy-harvesting performance across different radii reveals that, at a radius of 16 mm, the dual-beam system ensures satisfactory voltage capture across all frequency bands. In contrast, at a radius of 14 mm, the system demonstrated superior energy-harvesting performance, specifically in the 10–15 Hz band. The energy captured by the 16 mm radius configuration in the 10–15 Hz frequency band shows little difference from that captured by the 14 mm radius configuration. A comprehensive evaluation of the energy capture performance across all frequency bands indicates that the dual-beam system achieves optimal energy harvesting efficiency at a radius of 16 mm when the magnetic angle is 130°. Under this condition, the effective frequency bands of the vertical and cross beams are broadened by approximately 1.7 Hz and 2 Hz, respectively, significantly enhancing the wideband energy capture capabilities of the system.

### 4.5. Comparison of the Results of the Best Combination with the Control Group

Table 10 presents the results obtained by combining the two sets of variables for the experimental group. It can be observed that when the double beam is arranged with a radius of 16 mm and magnetic angle of 130°, the energy capture effect of the entire system is the best. Because the double girder was placed orthogonally, the voltage value captured by the double girder was squared and rooted, and the total voltage value obtained was approximately 15.329 V. Compared with the energy captured by the control group, the energy captured by the system in this state increased by 770%.

## 5. Conclusions

In this study, a vibration energy harvester based on a magnetically coupled cantilever beam in the orthogonal direction was designed, and the energy-trapping effect of the structural system was changed by adjusting the magnetic angle and magnetic radius. The optimal angle and distance of the double beam within 5–20 Hz were explored to improve the frequency-trapping efficiency of the entire system and achieve a widening of the frequency band. Experimental analysis shows that when the double beam is at a magnetic angle of 130° and radius of 16 mm, the system has the best energy capture effect. However, there are still limitations in this experiment. First, the parameter optimization range is limited; the magnetic angle and radius are only locally optimized in the 5–20 Hz frequency band, and the global frequency of bridge vibration is not extended to a wider frequency band. Second, the theoretical model is not well matched with the experiment, and the nonlinear dynamic equation of magnetic–mechanical–electrical coupling is not established, which leads to an insufficient explanation of the physical mechanism of parameter sensitivity. As an experimental exploration, this study has currently only completed experimental validation at the structural level. In the future, we will dedicatedly engage in systematic theoretical modeling and derivation with a rigorous attitude, aiming to improve the research framework of this structure and advance it from the experimental stage toward a complete framework integrating theory and practice.

## Figures and Tables

**Table 1:** Parameters of manufacturing physical models.

Parameter	Symbol	Numeric Value
The quality of magnets and plastic devices	$m_{s}$	0.00877 (kg)
Young’s modulus of a beam	E	90 (GPa)
The density of the beam	ρ	8470 ($kg/m^{3}$)
The size of the beam	L×B×H	100 × 20 × 0.5 (mm)
The size of the magnet	$L_{m}\times B_{m}\times H_{m}$	10 × 10 × 10 (mm)
Residual magnetic flux density of the magnet	$B_{r}$	1.21 (T)
The distance from the fixed block to the center of the circle	$D_{1}$	20 (mm)
The thickness of the gasket	$D_{2}$	2 (mm)
The length of the iron plate (5).	$D_{3}$	170 (mm)
The length of the iron plate (6).	$D_{4}$	250 (mm)
The shaker increases the speed	$V_{0}$	2 (Hz/min)
Piezoelectric element size	$L_{p}\times B_{p}\times H_{p}$	41.40 × 16.26 × 0.27 (mm)
The dielectric constant component at constant strain	$\varepsilon_{33}^{s}$	12 (nF/m)
Strain coefficient of the piezoelectric layer	$d_{31}$	23 × 10^−12^ (C/N)

**Table 2:** Effective voltage value captured by with a radius of 14 mm.

Constituencies	Angle (°)	Capture Voltage in the 5–10 Hz Band (V)	Capture Voltage in the 10–15 Hz Band (V)	Capture Voltage in the 15–20 Hz Band (V)	Constituencies	Angle (°)	Capture Voltage in the 5–10 Hz Band (V)	Capture Voltage in the 10–15 Hz Band (V)	Capture Voltage in the 15–20 Hz Band (V)
Vertical beam	90	0	4.377	4.543	Transverse beam	90	0	5.210	1.525
	100	0	4.816	4.606		100	0	5.339	1.542
	110	0	6.639	1.268		110	0	5.638	0
	120	0	6.732	0		120	0	5.564	0
	130	0	6.955	0		130	0	5.517	0
	140	0	7.308	0		140	0	5.077	0
	150	0	6.650	0		150	0	3.922	0
	160	0	6.800	0		160	0	3.142	0
	170	0.445	6.950	0		170	0	2.516	0
	180	0.480	7.466	0		180	0	1.778	0

**Table 3:** Effective voltage value captured by double girder with a radius of 16 mm.

Constituencies	Angle (°)	Capture Voltage in the 5–10 Hz Band (V)	Capture Voltage in the 10–15 Hz Band (V)	Capture Voltage in the 15–20 Hz Band (V)	Constituencies	Angle (°)	Capture Voltage in the 5–10 Hz Band (V)	Capture Voltage in the 10–15 Hz Band (V)	Capture Voltage in the 15–20 Hz Band (V)
Vertical beam	90	2.069	4.414	3.239	Transverse beam	90	0.897	3.103	1.555
	100	2.075	4.216	3.591		100	0.797	2.986	1.647
	110	2.001	6.207	4.062		110	1.083	4.653	1.849
	120	2.008	6.581	4.166		120	1.237	4.858	1.937
	130	2.256	6.866	3.832		130	1.549	4.986	1.662
	140	2.673	7.290	2.821		140	1.787	5.058	1.365
	150	3.100	7.936	0.332		150	1.944	5.230	0
	160	3.358	8.841	0		160	1.681	6.003	0
	170	3.455	7.198	0		170	1.188	4.674	0
	180	3.529	6.676	0		180	0	1.699	0

**Table 4:** Effective voltage value captured by double girder with a radius of 18 mm.

Constituencies	Angle (°)	Capture Voltage in the 5–10 Hz Band (V)	Capture Voltage in the 10–15 Hz Band (V)	Capture Voltage in the 15–20 Hz Band (V)	Constituencies	Angle (°)	Capture Voltage in the 5–10 Hz Band (V)	Capture Voltage in the 10–15 Hz Band (V)	Capture Voltage in the 15–20 Hz Band (V)
Vertical beam	90	0.733	4.359	5.081	Transverse beam	90	0	3.361	1.473
	100	0	4.168	5.645		100	0	3.676	1.409
	110	0	4.336	5.613		110	0	3.792	1.344
	120	0	4.197	5.342		120	0	3.849	1.218
	130	0	5.639	3.220		130	0	3.928	0
	140	0	5.887	1.699		140	0	3.664	0
	150	0	5.704	0.278		150	0	3.343	0
	160	0	5.664	0		160	0	2.866	0
	170	0	7.268	0		170	0	1.883	0
	180	0	7.065	0		180	0	1.023	0

**Table 5:** Effective voltage value captured by double girder with a radius of 20 mm.

Constituencies	Angle (°)	Capture Voltage in the 5–10 Hz Band (V)	Capture Voltage in the 10–15 Hz Band (V)	Capture Voltage in the 15–20 Hz Band (V)	Constituencies	Angle (°)	Capture Voltage in the 5–10 Hz Band (V)	Capture Voltage in the 10–15 Hz Band (V)	Capture Voltage in the 15–20 Hz Band (V)
Vertical beam	90	0	3.301	6.248	Transverse beam	90	0	3.598	1.272
	100	0	3.254	6.396		100	0	3.717	1.198
	110	0	3.091	6.125		110	0	3.451	0.858
	120	0	5.911	2.380		120	0	3.448	0
	130	0	6.043	0		130	0	4.787	0
	140	0	6.046	0		140	0	4.574	0
	150	0	6.663	0		150	0	4.120	0
	160	0	6.908	0		160	0	3.684	0
	170	0	7.029	0		170	0	3.300	0
	180	0	7.721	0		180	0	0	0

**Table 6:** Summary of parameter sensitivity.

Radius (mm)	Synthesis of Optimal Angles	Dominant Mechanism
14	100°	The negative stiffness effect under strong magnetic force
16	130°	Axial magnetic force compensates for distance—induced attenuation and enhances modal coupling.
18	90°	Orthogonal coupling maintains the potential energy gradient and suppresses chaos.
20	100°	Some axial components compensate for the weak magnetic force, balancing the stiffness and damping.

**Table 7:** Effective voltage value captured by angle 90 double girder.

Constituencies	Radius (mm)	Capture Voltage in the 5–10 Hz Band (V)	Capture Voltage in the 10–15 Hz Band (V)	Capture Voltage in the 15–20 Hz Band (V)	Constituencies	Radius (mm)	Capture Voltage in the 5–10 Hz Band (V)	Capture Voltage in the 10–15 Hz Band (V)	Capture Voltage in the 15–20 Hz Band (V)
Vertical beam	14	0	4.377	4.543	Transverse beam	14	0	5.210	1.525
	16	2.069	4.414	3.239		16	0.897	3.103	1.555
	18	0.733	4.359	5.081		18	0	3.361	1.473
	20	0	3.301	6.248		20	0	3.598	1.272

**Table 8:** Effective voltage value captured by angle 100 double girder.

Constituencies	Radius (mm)	Capture Voltage in the 5–10 Hz Band (V)	Capture Voltage in the 10–15 Hz Band (V)	Capture Voltage in the 15–20 Hz Band (V)	Constituencies	Radius (mm)	Capture Voltage in the 5–10 Hz Band (V)	Capture Voltage in the 10–15 Hz Band (V)	Capture Voltage in the 15–20 Hz Band (V)
Vertical beam	14	0	4.816	4.606	Transverse beam	14	0	5.339	1.542
	16	2.075	4.216	3.591		16	0.797	2.986	1.647
	18	0	4.168	5.645		18	0	3.676	1.409
	20	0	3.254	6.396		20	0	3.717	1.198

**Table 9:** Effective voltage value captured by angle 130 double girder.

Constituencies	Radius (mm)	Capture Voltage in the 5–10 Hz Band (V)	Capture Voltage in the 10–15 Hz Band (V)	Capture Voltage in the 15–20 Hz Band (V)	Constituencies	Radius (mm)	Capture Voltage in the 5–10 Hz Band (V)	Capture Voltage in the 10–15 Hz Band (V)	Capture Voltage in the 15–20 Hz Band (V)
Vertical beam	14	0	6.955	0	Transverse beam	14	0	5.517	0
	16	2.256	6.866	3.832		16	1.549	4.986	1.662
	18	0	5.639	3.220		18	0	3.928	0
	20	0	6.043	0		20	0	4.787	0

**Table 10:** Summary of the effect of energy capture.

Constituencies	Radius (mm)	Magnetic Angle (°)	Effective Voltage Value of Vertical Beam Capture (V)	Beam Capture Effective Voltage Value (V)	Vertical Beam Widening Band Value (Hz)	Crosshead Widening Band Value (Hz)
Experimental group	16	130	12.954	8.196	1.7	2
	14	100	9.423	6.881	1.5	1.5
	16	90	9.721	5.556	1	2.5
Control group	14	90	1.980	0		

## Data Availability

Data will be made available on request.
